# Shaping the Field: A Review of the Use of Theory in Research on Research Integrity

**DOI:** 10.1007/s11948-026-00587-y

**Published:** 2026-03-05

**Authors:** Marina Lambert, Lise Degn

**Affiliations:** The Danish Centre for Studies in Research and Research Policy, Department of Political Science, Aarhus University, Bartholins Allé 7, 1331, 019, Aarhus, C 8000 Denmark

**Keywords:** Research integrity, Theory, Review, Research misconduct, Meta research

## Abstract

Interest in research integrity (RI) has proliferated and gained prominence, particularly within the past decade – both in policy and academic environments. Research on RI might be considered an emerging research field, with dedicated journals and conferences. However, it is still characterized by fragmentation and is often carried out by researchers belonging to other fields (e.g. medicine, psychology, sociology, business, law, bioethics), thereby bringing varied approaches to the study of RI. The implications of this for the knowledge produced are, however, still understudied. Our study examines the place of theory in the shaping of the field of research on RI and how the choices of theoretical frameworks in given studies shape the logic of inquiry. To provide an inclusive overview of the relevant research and the theoretical variation underpinning it, we conducted systematic searches of SCOPUS, PubMed, and Web of Science (WoS) databases for English-language articles published between 2010 and 2023, based on a pre-defined set of search terms. The study finds that the theoretical landscape is highly heterogenous. It is, to some extent, dominated by grounded theory, personality psychology and institutionalism, but also engages a very broad scope of other theoretical perspectives, including social psychology, psychoanalytic theory, and narrative analysis. Through closer analysis of a group of studies that share focus on the relationship between pressure and research misconduct, this review elucidates the far-reaching implications that the choice of theory has on every aspect of an individual study and the field of research on RI overall.

## Introduction

Interest in RI has proliferated and gained prominence, particularly within the past decade – both in policy and academic environments (Aubert Bonn & Pinxten, 2019; Carcausto-Calla et al., 2023). Research on RI might be considered an emerging research field, with dedicated journals and conferences. However, it is still characterized by fragmentation and is often carried out by researchers belonging to other fields, thereby bringing varied approaches to the study of RI (e.g. medicine, psychology, sociology, business, law, bioethics). The implications of this for the knowledge produced are, however, still understudied. This review examines the place of theory in research on RI and how the choices of theoretical frameworks in given studies shape the logic of our inquiry into the issues of RI. Recognising and building on a vast pool of exploratory studies in the field, this review argues that systematic analysis of theoretical frameworks employed in research on RI is both timely and imperative for further advancement of the field. In providing a comprehensive overview of existing theoretical approaches developed and applied in the studies of RI, this review’s contribution is poised to advance our ability to better understand the dynamic of the field and the role of theory in its development.

## Research on RI as an Emerging Field

Research on RI arose from political and science systemic concern over misconduct cases, which sparked debate particularly in the US in the early 1980s (Roberts et al., 2020). With this debate and attention came institutions, most prominently the Office of Research Integrity in the US, and in Europe this institutionalization was reinvigorated in the early 2000s and 2010s. While the concern for the integrity of research is naturally a much older issue (as also demonstrated in Roberts et al., 2020), RI as a research topic or area in itself is a relatively new phenomenon. Several reviews of the content or knowledge claims within the area have emerged over the past years (e.g. Gross, 2016; Xie et al., 2021; Carcausto-Calla et al., 2023) but in this particular study we focus on the research area itself, and its advancement. As mentioned above, we approach research on RI as an emerging research field, following the definition by Cole (1983) as “*all work being done on a particular cognitive problem*”. Within science studies the debate around how to understand and analyze the epistemic and social organization of science is ongoing, and it is beyond the scope of the present review to determine whether the area is indeed an area, a thematically focused research community (Fagerberg & Verspagen, 2009) or a scientific field (Whitley, 2000). However, our argument is that in order for a research area to advance, it needs to advance theoretically as well, not just empirically. This review’s focus on theory is thereby informed by the value and unique capacity of theory “*to provide interpretive and/or explanatory insights into phenomena and the relations among them*” (Rule & John, 2015, p.2).

While it can be argued that theory in research is ubiquitous, it is not always evident. This is particularly true when considering the place of theory in research on RI. Collins and Stockton (2018) argue that even in cases where “*the exploratory nature of a study overrules the benefits of a theoretical framework, theory-free research does not exist*”, whereby a “*researcher who cannot articulate a theoretical framework may not have done the difficult and essential work to unearth their deepest operating principles and preconceptions about their study*” (p.2). This review will not undertake the task of “unearthing” the tacit and implicit principles underpinning each study in the field, but will rather focus on the analysis of those studies that recognise and engage theory explicitly. In the field of research on RI, as this review will demonstrate, the role of theory is still emergent, both in its practical use and in recognition of its merit. We argue that theory is what proliferates and opens up knowledge, enables a move from descriptive to explanatory, thus advances the field, promotes the relevance and pertinence of research within and far beyond academia (Saldaña, 2024).

There are many competing and complementary definitions of theory, which reflect the diversity of contributions theory offers to research. Thus, theory can be understood as a “*thinking tool we use in our attempt to explain human behaviour*”; a “*unified, systematic causal explanation of a diverse range of social phenomena*”; or an “*explanatory scheme comprising a set of concepts related to each other through logical patterns of connectivity*” (Saldaña, 2024, p.2). We define theory as “*webs of interlocking concepts that facilitate the organization of empirical material by providing explicit interpretive frameworks that researchers use to make their data intelligible and justify their choices and methodological decisions*” (Bendassolli, in Collins & Stockton, 2018, p.5). To further nuance our understanding of theory, we appeal to Saldaña’s six properties of a theory, which we find particularly helpful in guiding our analysis of the place of theory in the field of RI: 1) theory expresses a patterned relationship between two or more concepts; 2) it predicts and or manages actions through propositional logic; 3) it accounts for parameters of and/or variation in the empirical observations; 4) it explains how and/or why something happens, sometimes by stating its cause(s); 5) it suggests generalizability and/or transferability to related social contexts; and 6) it provides insights and/or guidance for improving social life (Saldaña, 2024).

Having outlined the operational definition of theory, the review will turn to the analysis of the role of theory in research on research integrity. The review is structured to map out the thematic variation across the selected studies, which comprises three main themes: RI, questionable research practices (QRP’s), and research misconduct (RM), as well as three supplementary themes that partially intersect with the above three main topics, namely, replication, co-authorship practices, and study retractions. These themes have emerged in consideration of empirical variation among the studies included in the review (see Annex 1 for the full list of included articles, theories employed and thematic clustering/attribution). The review further reflects on the diversity of theories employed across the studies, distinguishes different modes of theory application, and finally, analyses how a choice of theory shapes respective studies and potentially results in significantly diverging findings within the scope of same topic and similar research question.

## Methods

The studies in the focus of this review cover a variety of topics within the scope of research on RI. This section will discuss the selection process and inclusion criteria.

Firstly, to provide an inclusive overview of the relevant research and the theoretical variation underpinning the field of RI, we conducted systematic searches of SCOPUS, PubMed, and WoS databases for articles published in English between 2010 and 2023, based on a pre-defined set of search terms (that include: research, academic, and scientific integrity, research and scientific misconduct, good and questionable research practices). The initial search has produced 4.524 articles, where 2.920 articles were identified through Scopus, 2.164 articles retrieved via WoS (1.115 duplicates taken out); PubMed produced 2.073 results (subsequently 1.518 duplicates were taken out).

Secondly, based on the analysis of titles and abstracts of the 4.524 publications identified in the initial search, the review selected for further analysis of 472 articles specifically focused on research integrity, which included studies on scientific misconduct, questionable research practices, responsible conduct of research more broadly, as well as in their individual manifestations across different disciplines. A number of studies focusing on academic integrity, responsible research and innovation, research ethics, bioethics, ethical care of animals in research, clinical ethics, ethical role of universities in society, have been considered, deemed exceeding the scope of this review and excluded from the final pool. The review has included research articles only, omitting editorials, letters, committee recommendations, comments on policy statements, calls for guideline clarifications, and opinion pieces.

**Fig. 1:**
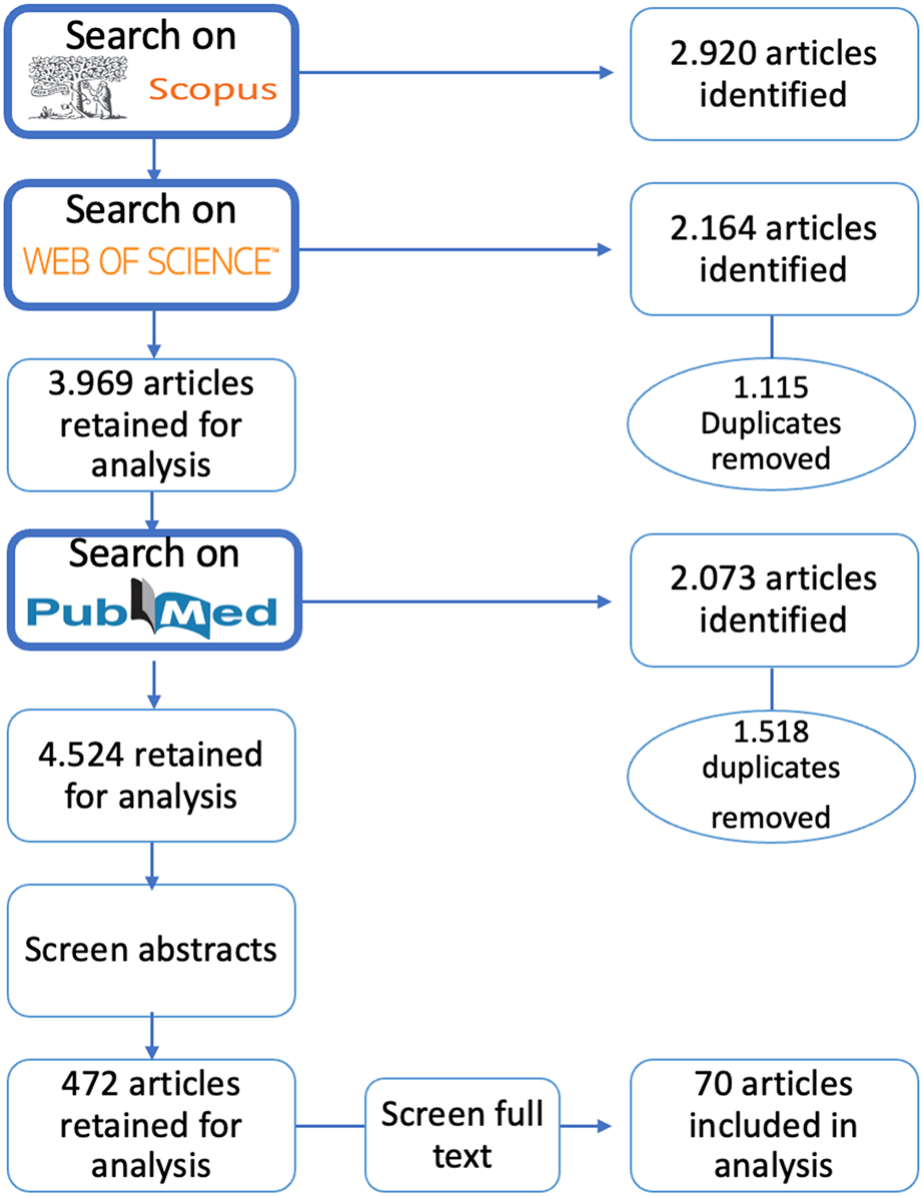
Study flow chart. Search terms: (TITLE-ABS-KEY (“responsible conduct of research”) OR TITLE-ABS-KEY (“responsible research practice”) OR TITLE-ABS-KEY (“scientific misconduct”) OR TITLE-ABS-KEY (“research misconduct”) OR TITLE-ABS-KEY (“research integrity”) OR TITLE-ABS-KEY (“academic integrity”) OR TITLE-ABS-KEY (“scientific integrity”) OR TITLE-ABS-KEY (“research fraud”) OR TITLE-ABS-KEY (“scientific fraud”) OR TITLE-ABS-KEY (“questionable research practices”))

Thirdly, full texts of the selected 472 articles were analysed and classified according to whether they explicitly engaged one or more theories in its approach to the analysis of RI. Recognizing a growing body of atheoretical (non-theory-driven) research that advocate for the promotion of RI (e.g. Bouter, 2020), develop classifications of types of RM (e.g. Shaw, 2019), and propose approaches to awareness-raising in the research community (e.g. Satalkar & Shaw, 2019), this review excluded purely empirical (e.g. Christopher, 2022), comparative (e.g. Rabelo et al., 2020) and descriptive analyses (e.g. Ripley et al., 2012), and focused strictly on the studies whose design built on employing various theories or elements of theoretical frameworks as a principal point in guiding their analytical work (*N* = 70) (See Fig. 1). More specifically, this review sought to include all studies that engaged one or more theories in their analysis, irrespective of specific approach to their conceptualisation of theory, or the mode of theory application. This review will analyse in detail the diversity of theoretical approaches engaged in the field by explicitly accounting for the variation of theories, their conceptualisations and modes of application across the selected studies. In so doing this review seeks to contribute to the discussion of the role of theory in research on research integrity.

## First-Order Analysis: Categorising Research on RI

Seeing as “theory is in the eyes of the beholder” and one researcher’s view of theory and claim to its application may not be universally agreed upon (Saldaña, 2024, p.4), in classifying a study as employing a theory in its analytical work, we recognize diverging conceptions of theory across the reviewed studies, different extents of theory engagement, as well as alternative modes of theory application. A closer consideration of these divergencies and their impact on our understanding of the role of theory in research on RI constitute the focus of this review.

### Thematic Categorisation

The studies included in this review were initially subjected to what we might call a first order categorisation. Having identified the theory-driven (or theory-informed) studies, this categorisation addressed the empirical variation in the selected studies, which was captured in six thematic clusters. The six themes recognised here are emergent and are based on the topics and empirical foci of the studies included in the review.

**Fig. 2:**
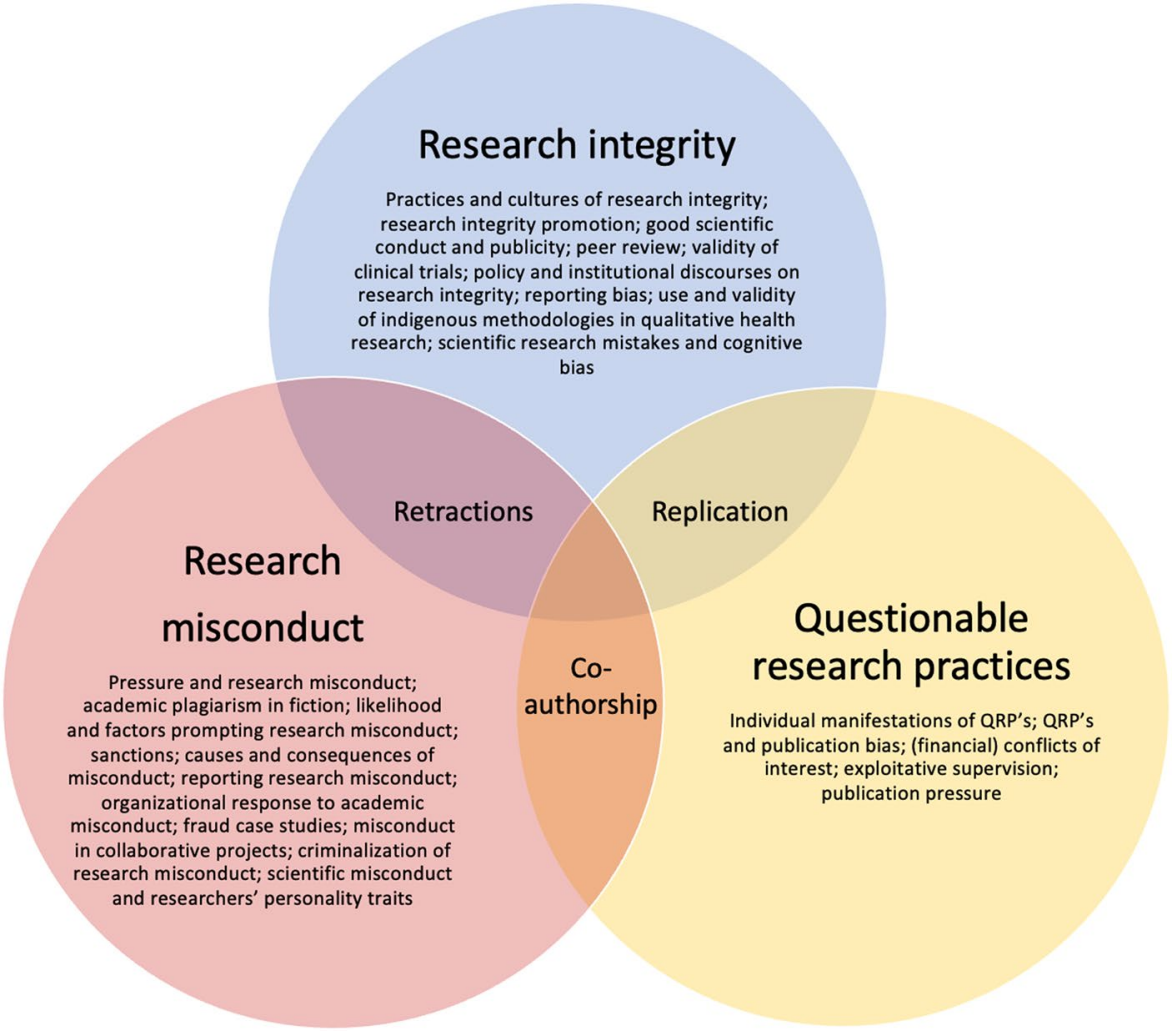
Thematic variation in research on research integrity

Most of the articles addressed the issues of research integrity (16 articles), research misconduct (36 articles), and questionable research practices (11 articles), which formed three core themes; while fewer articles focused specifically on the topics of problematic co-authorship practices (nine articles), retracted research (seven articles), and replication crisis (three articles), which intersect across the three core themes and formed further three (less prevalent, but distinct) themes (see Fig. 2). Moreover, each core theme captured a variation of topics that addressed broader or more nuanced topics within the scope of RI, research misconduct, and QRP’s respectively. This thematic variation reflecting the complexities entrenched in the field of research on research integrity is depicted in Fig. 2 below:

This review’s delineation between the themes rests on the conceptions of ‘research integrity’, ‘questionable research practices’, and ‘research misconduct’ as stated by the studies’ authors. This review refrained from qualifying or reconciling discrepancies in definitions of QRP’s, RM, and RI. In cases where the same article elaborated on more than one theme, its attribution rested on its primary focus. In cases where two or more themes were prominently represented, a given study was considered iteratively in the analysis of all relevant thematic categories (see Annex 1 for the list of themes and articles attributed to each theme).

### Theoretical Categorization

In our analysis of full texts of the 472 articles included for thematic fitness, we have proceeded with the selection of articles that engaged one or several theories in their analysis, noting on varying approaches to authors’ definitions of theory, distinct modes and extents of theory application. Namely, in our final round of analysis we have included all articles (N70) that employed one or more theories, or elements of theoretical frameworks in their analyses, irrespective of the variation among the theories, theoretical approaches, analytical frameworks, or differences in the approaches to their use in the analyses. The different dimensions in the variation in the use of theory in research on research integrity will now be considered in detail.

Firstly, the diversity and heterogeneity of theories engaged in the field is significant. Among theories most prominently represented are ‘rotten apples’ theory of misconduct (e.g. Paradeise & Filliatreau, 2021), personality psychology (e.g. Tijdink et al., 2016), grounded theory (e.g. Nelson et al., 2020), and Scandinavian institutionalism (e.g. Czarniawska, 2009). However, the field also engages a broad scope of theoretical choices that are less prevalent, and which distinctly hail from other areas of social science research, such as psychoanalytic theory (e.g. Zwart, 2017), critical discourse analysis (e.g. Davies, 2019), and social identity theories (e.g. Stürmer et al., 2017) (see Annex 1 for the full list of reviewed studies and theories they employed).

Secondly, there is a considerable variation in the conceptualisation of theory across the selected studies. “*Theories come in all shapes and sizes*” (Richards in Saldaña, 2024, p.7), to which this review attests. Reflecting on the diversity of theories employed in research on RI, we recognise their fitting with the established three-tier view of theories: the “little t” theories (ones that theorize about a specific case, and are also known as local, substantive, micro theories or idiographic theories), the “BIG T” theories (which reach far beyond a single case, attempt to “transcend bounded contexts such as populations, settings, and time”, have claim to universality of application, and are often referred to as formal, general, grand, macro theories, metatheories or nomothetic theories), and the mid-range (also referred to as practitioner or experiential), which cover all that are in between (Saldaña, 2024). This diversity in range is exemplified by variation - from rational choice theory (e.g. Gordon, 2014) to cultural theory of punishment (e.g. Hesselmann, 2019b); while some of the studies relied on theorizations, rather than theories (such as Hardwig and Frost-Arnold theorizing (Andersen, 2014), as well as analytical approaches and conceptual frameworks (such as “conceptual frame of norms and maxims” (Meyer & Sandøe, 2012), and McGuire’s perspectivist approach to knowledge construction (Świątkowski & Dompnier, 2017). Table 1 reflects the variety of theories involved and shows their distribution across the studies in the three core and three supplementary themes.

**Table 1:** Distribution of theories and analytical frameworks across the six thematic clusters (see Annex 1 for distribution of the 70 analysed articles across the six themes and references to theories and/or analytical frameworks employed in each study)

Theme	Theories
Research integrity	Bourdieusian sociology; social epistemology; post-colonial theory and indigenous epistemology; organisational theory/new institutionalism; organisational justice theory, open-systems theory, dual-process theory, reflexive thematic analysis, grid-group cultural theory, (grounded theory);‘ecological’, ‘person-centred’ and ‘strength-based’ analytical lenses; policy analysis framework; concept of norms and maxims, Merton theoretical framework/“Desire-Belief-Opportunity” (DBO) model; Orders of worth (Boltanski and Thevenot), language game (Wittgenstein), moral economy of science (Datson); theorisation of peer review and retractions; Parson’s theoretical framework.
Research misconduct	Lacanian psychoanalysis, Foucauldian perspective; personality psychology, personality traits, Dark Triad (narcissism, psychopathy, Machiavellianism); psychology (in broader sense); post-colonialism, discourse analysis; gender theory; Rational choice theory, general strain theory, cultural theory, network theory, bounded rationality theory of misconduct; Prospect theory, new public management, criminological theory (and deviance theories), corruption theory; rotten apple theory of misconduct; Sovacool’s three theories of misconduct; ‘Stressful theory’/‘Imperfect environment theory’; resource dependence theory, theory of social power; game theory; economic theory; labelling theory; theory of social stigmatisation; organisational justice theory; criminology theories; situational action theory in criminology; theory of sensemaking, cultural theory of punishment; grounded theory (theme analysis); concept of boundary work, Latour’s perspective; Bourdieu’s ‘academic capital’; Merton/Matthew effect; Hardwig and Frost-Arnold theorising; concept of social-cognitive mechanisms
Questionable research practices	Bad apple theory, general strain theory, organisational culture theory; neo-institutional theory; behavioural biology, psychology; personality psychology (dark triade); social identity, social representation theory; economic theory; situational action theory in criminology; theory of communicative action; Foucauldian perspective; goal standard model
Retractions	Rational choice theory, corruption theory; rational crime theory (“economics of rational crime” framework); social cognition; concept of boundary work, Latour ‘s perspective; Bourdieu’s academic capital, Merton/Matthew effect; Benford’s law; labelling theory, concept of visibility; theorization of peer review and retractions
Co-authorship	Grounded theory, (theme analysis); decision theory; theories of inequalities; theory of social stigmatisation; Habermas and Merton theorising. Hardwig and Frost-Arnold theorising; concept of social-cognitive mechanisms; concept of boundary work, Latour’s perspective
Replication	Gender theory, personality psychology, personality traits (impulsiveness, aggressiveness, Machiavellianism etc.); Matching law; McGuire’s perspectivist approach to knowledge construction

The third finding of the theoretical categorisation was that the application of theory, i.e. the ways in which theories lend themselves to the analysis, are also considerably varied. The variation in the conceptualisation of theory in the research on RI is reflected in the discrepancies between studies’ claims to their use of theory and theory’s actual application in the analyses. This review finds a three-gear approach to theory engagement emerging from the evaluation of the diverging conceptions and alternative modes of application of theory in the studies considered here: 1) use of one or several theories/theoretical approaches stated explicitly within the discussion of the research design and consistently carried out within the analytical part of a given study (exemplified by Zwart, 2017); 2) a theorisation carried out alongside the analysis and discussion (e.g. Schoenherr, 2015); and 3) an operationalisation of one or several concepts linked to a theoretical framework not fully engaged in the study, but used to functionally guide the analytical work carried out in the study (e.g. Paruzel-Czachura et al., 2021). This variation in the use of theory in the field of research on RI reflects (at least in part) Collins and Stockton’s (2018) three modes of theory application established and recognised in qualitative research: 1) theory of research paradigm and method; 2) theory building as a result of data collection, and 3) theory as a framework to guide the study. Moreover, it is also worth noting on a small group of studies that contained a claim to use a theory (at introduction or conclusion), but have not engaged said theoretic approach in either analysis or discussion sections of the study (e.g. Parker et al., 2022). To elicit another dimension of nuance entailed in this discussion, we can address the case of grounded theory. Included in this review we find a group of studies, which have employed grounded theory in combination with one or more theories (e.g. Valkenburg et al., 2021) or relied on it solely in guiding their analytical work (Street et al., 2010). The critique of grounded theory beyond the field of research on RI is equally pertinent for the purposes of this analysis - the term’s liberal application “to label any research endeavour that involves coding, any form of qualitative data analysis, and any kind of theory construction” amounts to grounded theory being a “shorthand for qualitative research and the lack of a well-articulated analytical strategy” (Timmermans & Tavory, 2012, p.168). This critique of grounded theory reflects the potential of discrepancy between the formal claim to theory engagement and its actual use in the analysis and should be recognised as relevant beyond the studies reliant strictly on grounded theory, but valid in general consideration of the use of theory in the research on RI. Table 1 shows the distribution of different theories and various analytical frameworks (or their elements) across the core and supplementary themes considered in this review.

Another level of variation in the use of theory in the field can be discerned in diverse analytical functions through which theory was found to contribute to the reviewed studies, which includes (but is not limited to) informing hypotheses, interpreting findings, developing models, creating taxonomies and classifications, and providing definitions and clarifications to concepts.

## Second-Order Analysis: The Role of Theory

Having mapped out what theoretical diversity in the field of research on RI entails, this review takes a step further to consider the role of theory in research on RI by scrutinising a small group of studies selected for their specific focus on same topic within the field. By closely considering the group of studies that address same, similar or compatible research question, the following section specifically teases out the role of theory and the impact that diverging choices of theory and its application has on the field of research on RI.

### Pressure as Cause of Research Misconduct

The group of studies considered here stems from the core theme ‘Research misconduct’ and focuses on the topic of ‘pressure as cause of research misconduct’. Being the most populated group of theory-driven studies that share an empirical focus and present with a similar research question, it offers best vantage point to closely inspect the role of theory and variation of different theoretic approaches in research on RI. This group comprises eight studies that address the somewhat controversial status of causal links between pressure and RM. Moreover, the selected studies demonstrate considerable variation of theoretical approaches employed in the analytical work undertaken across the studies, as well as offer insight into different modes of conceptualising and operationalising theory as such (see Table 2).

**Table 2:** Studies focused on the topic “Pressure as cause of research misconduct”

Study	Research question	Theory	Mode of application
Lacetera and Zirulia (2011) The economics of scientific misconduct	Why scientists commit fraud and how fraud can be detected and prevented?	Game theory	Theory used to develop a model (for predicting research misconduct)
Hall and Martin (2019) Towards a taxonomy of research misconduct: The case of business school research	What research behaviour is deemed appropriate/inappropriate; which stakeholders it affects; which pressures and incentives likely to exacerbate such misconduct?	Rational choice, strain, cultural, network, and bounded rationality theories of misconduct	Theory is used to create a theoretically based taxonomy
Holtfreter et al. (2019) The perceived causes of research misconduct among faculty members in the natural, social, and applied sciences	What are the perceived causes of research misconduct among faculty members in the natural, social, and applied sciences?	Criminology theories: ‘bad apples’ approach – personality psychology; ‘bad barrel’ approach – general strain theory	Theories used to elucidate different dimensions of causal connections between pressure and research integrity
Paruzel-Czachura et al. (2021) Publish or be ethical? Publishing pressure and scientific misconduct in research	What is the association between publication pressure and researchers’ dishonesty?	Social psychology	Theory lends concepts to discuss findings
Heuritsch (2021) Reflexive behaviour: How publication pressure affects research quality in astronomy	To what extent is research (mis-) behaviour reflexive, (i.e., dependent on perceptions of publication pressure and distributive and organisational justice) and what impact does scientific misconduct have on research quality?	‘Bad apple’ theory; general strain theory, organisational culture theories, new public management, rational choice theory	Theory informs hypotheses
Haven and van Woudenberg (2021) Explanations of Research Misconduct, and How They Hang Together	How do different explanations of research misconduct relate to one another and are they compatible?	Rational choice theory, ‘bad apple’ theory, general strain theory, prospect theory, organisational justice theory, new public management	Theories (as object) in focus of inquiry into limitations to their exploratory potential and compatibility
Huistra and Paul (2022) Systemic Explanations of Scientific Misconduct: Provoked by Spectacular Cases of Norm Violation?	Did systemic explanations of misconduct emerge in response to high-visibility cases of scientific norm violation?	Sovacool’s typology: ‘individual impurity’ (bad apples), ‘institutional failure’ (bad barrels), and ‘structural crisis’ (bad science systems)	Theory as object of inquiry
Yeo-Teh and Tang (2022) Perceived publication pressure and research misconduct: should we be too bothered with a causal relationship?	What is the status of causal relationship between perceived publication pressure and research misconduct?	Personality psychology, situational action theory in criminology	Theory elucidating limitations of existing/conventional theories of research misconduct

Hall and Martin’s (2019) inquiry into pressures and incentives encouraging RM engages theory by surveying several different theoretical approaches applied to analysing organisational misconduct. They make use of theory (a selection of theories) to develop a detailed comprehensive taxonomy that differentiates appropriate conduct, QRP’s and RM. This approach to the use of theory in research on RI aims to deliver clarifications to concepts and definitions developed on the basis of different theories surveyed in the study.

The study presents consequent application of five theoretical lenses - rational choice, strain, cultural, network and bounded rationality, and by unpacking the five approaches and the variation in their explanatory capacities for addressing pressures contributing to RM, the study yielded two-fold result. Firstly, it presented a classification of established theoretical approaches utilised in the analysis of organisational misconduct, which extended the argument for their pertinence in the research on RI and RM. Secondly, it demonstrated an alternative mode of theory application – it exemplified the utility of theory (concurrent use of a set of theories) in offering conceptual clarity.

Huistra and Paul (2022) focus on the origins of systemic explanations of scientific misconduct and consider the role of high-visibility cases of RM. The study employs Sovacool’s typology of explanations for scientific misconduct: ‘individual impurity’ (bad apples), ‘institutional failure’ (bad barrels), and ‘structural crisis’ (bad science systems). It engages with theory in an alternative way, namely by reflecting on the shift in the preferred theoretical paradigms employed in the emerging analyses of scientific misconduct cases. By focusing on several high-profile cases of RM, the authors discern a pattern indicating a transition toward “systemic explanations”, which they characterised as an increasingly prevalent approach to explaining RM.

Haven and van Woudenberg (2021) consider alternative explanations of RM and their compatibility, by focusing on the analysis of a single high-visibility case of RM (the Diederik Stapel case) through six different theories: Rational Choice theory, Bad Apple theory, General Strain Theory and Prospect Theory, Organisational Justice Theory, and New Public Management. The study branches out to explore the link between pressure and misconduct, insofar as several of the theories it applies recognise and maintain the causal link between the two. By consequently applying the six theories to the analysis of the Stapel case, the study argues that application of these theories offers partial explanations of RM. Moreover, it argues that in order to adequately carry out analysis and apply theory in the analysis, each theory presupposes existence and availability of specific data or evidence. Finally, the study found that four of the theories are compatible with one another, and it can be further argued that engaging several theories could be beneficial to reach comprehensive explanation of RM.

Lacetera and Zirulia (2011) inquire into the reasons behind scientific fraud and make use of game theory to zoom in on the role of publication pressure. Their analytical approach yields an alternative interpretation of the causal links between publication pressure and RM. They suggest that publication pressure rather curbs scientific misconduct – “a “publish-or-perish” paradigm may in fact reduce, and not increase, scientific misconduct” due to additional scrutiny from peers that must arise during the publication process (p.568). The use of theory is likewise somewhat alternative – the authors employ game theory to develop a model for research and publication process with the view to analyse the causes of scientific misconduct (supplemented with suggestions for detection and prevention). The study proposes a typology that distinguishes types of research that are more likely to be fraudulent, as well as types of scientists that are more likely to commit fraud.

Paruzel-Czachura et al. (2021) focus on self-reported publication pressure and self-reported scientific misconduct in research. Their study employs theory by engaging a selection of concepts to drive the analysis of the relationship between perceived publication pressure and scientific misconduct. The concepts “point-mania”, “impactophrenia”, and “pointosis” link to the broader framework of social psychology and are used to reflect on perceived psychological pressure as a cause for QRP’s. Thus, publication pressure is viewed as a form of psychological stress, which is linked to diminished ethical decision making, risky behaviour and scientific misconduct.

Yeo-Teh and Tang (2022) likewise question the causal relationship between publication pressure and QRP’s and scientific misconduct. They address the body of existing research that inquires into causal connection between pressure and misconduct (including the articles considered in present review) to point out their diverging findings as a point of departure for their own analysis of the entailed complexities. They argue that it is inherently difficult to establish direct causal relationship between perceived publication pressure and QRP/RM, because the former is a complex biopsychosocial construct that is variedly influenced by multiple personal and environmental factors. The authors appeal to personality psychology, consider personality traits (such as Machiavellianism), and call upon situational action theory in criminology theory to elaborate on the multi-factorial nature of RM to evaluate the complications arising in the attempts to settle ambiguities in the question of causality.

Heuritsch’s (2021) inquiry into the ways scientists respond to hypercompetition and publication pressure is contextualised in the studies of RI, organisational studies, and reflexive metrics. The article assesses role-associated factors, cultural aspects, and publication pressure in their capacity to explain the variance in perceived research misbehaviour and its impact on research quality in the field of astronomy. The study utilised five theories/theoretic approaches (Bad Apple theory; General Strain theory, Organisational Culture theories, New Public Management, Rational Choice theory) to devise and test six hypotheses aimed to explore the connection between perceived publication pressure and RI. The study employs theory to build a structural equation model to grasp the connections between role-associated factors (e.g. academic position) with environmental factors (e.g. perceived publication pressure and distributive and organisational justice), and the scientific misconduct and research quality.

Holtfreter et al. (2019) look at various conditions perceived to lead to RM. The study seeks to ascertain the extent to which known criminogenic factors contribute to RM, and finds that pressure to secure external funds, publish in top-tier journals, as well as low probability of detecting misbehaviour, are among the top perceived causes of RM. Conceptualisation of RM as white collar crime is framed through the prism of criminology theories. The article provides extensive consideration of different theories in their explanatory capacity relating to RM, whereas its focus is placed on exploring the impact from alternating attribution of rule-violating behaviour to individual or environmental factors. Thus, the consideration of the “Bad Apples” approach called upon personality psychology (Gottfredson and Hirschi’s theory), which zoomed in on low self-control as key personality trait linked to criminal and deviant behaviour, while social learning theory informed the argument that “rule-violating behaviour is the product of learned motivations and rationalisations” (p.2164). The “Bad Barrel” approach in the analysis of RM was considered through General Strain theory, which postulates that “deviance is a product of strain and stress that, if individuals are unable to cope with effectively, will result in negative emotions – anger, depression, anxiety, and malicious envy – that create pressure for corrective action” (p. 2164). Moreover, Deterrence theory framed the argument that “scholars considering scientific fraud will be deterred from doing so if the likelihood of apprehension is high (certainty principle), the punishment associated with doing so is sufficiently punitive (severity principle), and that such matters are dealt with swiftly (celerity principle)” (p.2164).

### Diverging Uses of Theory

The articles comprising this group demonstrate significantly diverging approaches in addressing a shared research question that scrutinises causal links between pressure and RM, and do so primarily through their divergent use of theory. Thus, Hall and Martin (2019) engage Rational Choice, Strain, Cultural, Network, and Bounded Rationality theories of misconduct to argue for a robust causal connection between pressure and misconduct. They use the above theories as alternative approaches to demonstrate, justify and explain the nature of this causal link, as well as illustrate how these different theoretic approaches unpack different aspects of this causal relationship. Complementary use of Rational Choice, Strain, Cultural, Network, and Bounded Rationality theories of misconduct collated to produce a theoretically based taxonomy of misconduct, pinpoints different sources of pressure and alternative levels of impact on the researcher, different pathways to impede research quality, as well as posits alternative explanations of the mechanisms establishing causality between pressure and RM.

Holtfreter et al. (2019) recognise a causal link between pressure and RM by viewing it through the prism of criminology theory. They use four theoretical approaches in the analysis of RM to discuss two diverging logics in the conceptualisations of RM resulting from pressure – by allocating responsibility and rooting agency at the level of an individual (researcher) or a system (institution).

The consideration of these two rationales – encapsulated through consequent scrutiny of the selected theories - was compelled to explore possible variation between researchers’ perceptions of conditions leading to RM across different disciplines. The study makes use of theory (theories) to explore different tier mechanisms that underpin how pressure prompts RM.

Furthermore, Paruzel-Czachura et al. (2021), also recognise the causal nature of relationship between pressure and misconduct, while maintaining a more tenuous connection to the social psychology framework by engaging several concepts to reflect on and contextualise the study’s empirical findings. Finally, Heuritsch (2021) calls upon Bad Apple theory, General Strain theory, Organisational Culture theories, New Public Management, Rational Choice theory, to design and test six hypotheses that directly and indirectly postulate causal connection between pressure and RM. Having used theories to propose the hypotheses, Heuritsch found those explicitly focused on perceptions of pressure resulting in misconduct to be upheld.

Nonetheless, Lacetera and Zirulia (2011) arrive at a contrary conclusion and argue that (publication) pressure ensures higher quality of research, which must result from an inherently more stringent scrutiny of scientific work. Their use of Game theory did less to guide the analysis as such, but rather involved instrumentalising theory to produce a model predicting RM and a typology of researchers likely to offend. Whereas exploration of the causal link between pressure and misconduct presents as one of several objectives in the study, having utilised theory to produce the model and the typology, it presents a distinct use of theory in research on RI, as well as leads to starkly diverging conclusion that denies the existence of causal connection between pressure and misconduct.

Furthermore, Yeo-Teh and Tang (2022) address the connection between publication pressure and RM (and QRP’s) to highlight the complexity of the processes involved and argue that any claim confirming or rejecting causal link between the two would be problematic and inconclusive. Reflecting on previous research that questions and disagrees on the status of causal links between pressure and misconduct, this study invokes theory to not merely provide an analytical framework for the analysis of the empirical data, but to attempt an explanation of the existing conflicting findings that had resulted from earlier analyses. It can be viewed as a use of theory in illuminating the inherent limitations to the existing uses of theory in the research on RI (focusing on the studies of causal connection between pressure and RM).

Moreover, Haven and van Woudenberg (2021) study can be seen as contributing to a similar objective. Their use of a set of theories in the analysis of a case of RM makes a two-fold contribution of particular interest to this review. Firstly, they offer a study that carries out consequent analysis of RM based on application of six different theories. Secondly, they elucidate the value of theory in the research on RI by methodically demonstrating the scope of variation in (and inherent limitations to) a study’s findings that result from researcher’s choice of theory.

It is also worth noting that the above group of studies further reflects another facet of variation in conceptualising theory. Namely, “Bad Apples” and “Bad Barrels” have been viewed as either full-fledged theories of RM, invoked as umbrella terms for groups of theories based on their alternating focus on an individual or an institution in underpinning the causes of misconduct, or problematising them not as theories, but as approaches reflective of the state of the field of research on RM more broadly, as well as viewed as narratives of research misconduct (Sovacool, 2008).

Our analysis demonstrates that different explanatory potentials entailed in the above theories lead to different conclusions and prescribe (or not) different approaches to tackling pressure as the cause of RM. These range from general contemplation of the approaches to influencing the research/institutional culture, to specific recommendations for amending individual field’s research practices. Application of different theoretical lenses presents in the analysis with diverging logics of prioritising some aspects of the studied phenomenon and dismissing others. The analysis of the group of studies focused on pressure and RM demonstrates how this divergence manifests in practice. As such, a choice to engage organisational culture theories places emphasis on the role of institutions, primes to consider environmental factors in the explanations of misconduct and limits the considerations of the role of individual researcher, whereas a choice to engage personality psychology reverses the focus and the logic of arriving at an explanation.

We can conclude that the studies that argue for or confirm a causal link between pressure and misconduct employ different constellations of theories to guide analysis resulting in their findings. Variation in theories further reflects different conceptions of pressure, its components and their modes of impeding research quality (mechanisms through which they promote RM). The studies that deny a causal link between pressure and RM or argue against the pertinence of a definitive stance on the question of causality, further demonstrate the impact that choice and mode of application of a theory in a study have on the study’s findings. Having closely considered the studies focused on pressure and RM, this review demonstrates how theories can be used to prove and disprove the same claim – that pressure leads to misconduct. In so doing, the review demonstrates how choice of theory in research on RI is crucial in shaping individual studies at every stage of the research process.

### Example of Misconduct in Collaborative Projects

The review’s findings are not limited to the studies of pressure and RM. The analysis of other (albeit less populated) groups of studies that share similar topics and research questions displays similar diversity of theoretical approaches and variation in the mode of theory conception and application. For example, studies centred around misconduct in collaborative projects (see Table 3), present the use of theory aimed at developing an analytical framework (Schoenherr, 2015); designing hypotheses (Mongeon & Larivière, 2016); conceptualising a phenomenon/practice (stigmatisation by association) (Hussinger & Pellens, 2019); theorising a concept (trust) (Andersen, 2014). The mode and degree to which a given study engages a theory varies likewise, ranging from merely lending a concept to the analysis (Mongeon & Larivière, 2016; Andersen, 2014) to collating several theories to promote an application of an additional/alternative theoretic approach within a field (Schoenherr, 2015).

**Table 3:** Studies focused on the topic “Misconduct in collaborative projects”

Study	Research question	Theory	Mode of application
Andersen (2014) Co-author responsibility: Distinguishing between the moral and epistemic aspects of trust	Is a co-author’s failure to identify the misconduct of a collaborator a moral failure that qualifies as scientific misconduct, or is it an epistemic failure that qualifies as poor science?	Hardwig and Frost-Arnold theorisation of trust	Theorisation of concept
Schoenherr (2015) Social-cognitive barriers to ethical authorship	What is the role of social-cognitive mechanisms in research misconduct?	Theorisations of social-cognitive mechanisms	Developing analytical framework
Mongeon and Larivière (2016) Costly collaborations: The impact of scientific fraud on co-authors’ careers	How do retractions in biomedical research affect co-authors’ research careers?	Merton’s theory (of cumulative advantage and disadvantage), concepts of Matthew’s effect, and Bourdieu’s symbolic capital	Theory informs hypothesis and explains findings
Hussinger and Pellens (2019) Guilt by association: How scientific misconduct harms prior collaborators	How does scientific misconduct harm prior collaborators?	Theory of social stigmatisation	Theory used to conceptualise a phenomenon and develop/reconcile existing ‘explanatory mechanisms’ applied in the field

Finally, having scrutinised the role of theory in shaping individual studies and the field of research on research integrity itself, it is important to note on the intricate connections between theory, data and data availability. Certain types of data lend themselves more willingly to some theories, while other theories require certain specific types of data, which may in turn result in various limitations across different study designs, as well as contribute to the variations in the logics of analyses and interpretation of results even among studies with similar empirical focus.

## Conclusions

In general, the present review has demonstrated that the field of research on RI is remarkably heterogeneous, both empirically and theoretically. Moreover, theoretical diversity presents in three distinct ways. Firstly, while only a small proportion of articles reviewed here were found to be engaging theory in their analyses (approx. 15%) (see Fig. 1), the field presents a considerable theoretical variation, both in terms of theory range and its field of origin. Secondly, the extent to which theories are engaged in the analytical work carried out in the studies considered in this review diverges in several distinct ways. Namely, theory can be found to systematically guide studies’ analytical work, be co-constructed alongside the discussion of studies’ empirical findings, or merely lend one or several concepts to a study’s analytical framework. Thirdly, the field is marked by considerable variation of the mode of theory’s application. The latter includes a broad scope of functions that theory performs within a study, which ranges from proposing the explanation to study’s findings to developing predictive models.

This review supports the argument that theory ensures rigor in research (Grant et al., 2018). Capitalising on theory’s explanatory potential allows us to elevate research from descriptive to analytical, whereby insufficient commitment to theory in research could be equally detrimental as insufficient commitment to method and result in a “simplistic product that lacks insights and is potentially irrelevant or not even considered knowledge production” (Collins & Stockton, 2018). Furthermore, “while explorative research seeks to find something “new,” theory-driven research seeks to elaborate on already known and hence predictable effects”. To paraphrase Burghardt and Bodansky (2021), maturation of the field of research on RI requires a move away from “the innovation requirement” toward theory-driven research, whereby “precise theorising needs to substitute novelty”.

The review reflects on how (sparsely) theory is used in research on research integrity, how diverse those theories used are, and how the choice of theory and its application ultimately impact the outcome of an individual study and stands to contribute to shaping the filed itself.

Having problematised the role of theory in research on research integrity, this review did not address the question of when descriptive is enough. However, this could present an interesting course for further analysis, namely, by surveying the remaining 85% of the studies that this review has in the earlier rounds of analysis identified as a-theoretical, to provide an overview of “when descriptive is enough”. Another possible course to advance our understanding of the role of theory in research on RI is to consider the backgrounds and affiliations of the authors of the 472 articles (including those who did not engage theory in their analyses) to get a full understanding of possible connections between authors’ disciplinary background and proclivity to employ theory in studies of research integrity.

In demonstrating how choice of theory shapes every aspect of research, from the logic underpinning study design to the interpretation of its findings, this review argues that consideration of theory in the research on RI is essential and timely, and further efforts to promote the role of theory in the field are imperative for its advancement.
